# Comparison of vaginal natural orifice transluminal endoscopic surgery and vaginal hysterectomy for benign indications: a prospective randomized controlled study

**DOI:** 10.1590/1806-9282.20250948

**Published:** 2025-12-05

**Authors:** Gazi Yıldız, Esra Keles, Pınar Yıldız, Murat Levent Dereli, Mehmet Mete Kırlangıç, Kasım Turan, Bahadır Alper Sargın, Emre Mat

**Affiliations:** 1 University of Health Sciences Turkey, Kartal Lütfi Kırdar City Hospital, Department of Obstetrics and Gynecology – Istanbul, Turkey.; 2 University of Health Sciences, Kartal Lütfi Kırdar City Hospital, Department of Gynecologic Oncology – Istanbul, Turkey.; 3 Umraniye Training and Research Hospital, Department of Perinatology – Istanbul, Turkey.; 4 University of Health Sciences, Ankara Etlik City Hospital, Department of Obstetrics and Gynecology, Division of Perinatology – Ankara, Turkey.; 5 Private Clinic, Department of Obstetrics and Gynecology – Van, Turkey.

**Keywords:** Natural orifice transluminal endoscopic surgery, Gynecologic surgeries, Pain, Vaginal hysterectomy

## Abstract

**OBJECTIVE:**

The aim of the study was to compare the surgical outcomes of vaginal natural orifice transluminal endoscopic surgery and conventional vaginal hysterectomy for benign gynecological conditions.

**METHODS:**

A prospective randomized controlled trial was conducted at a tertiary hospital from January 2025 to February 2025. Patients aged 40–80 who underwent hysterectomy for benign indications were included. Outcomes assessed included surgical outcomes, visual analog scale pain scores, and complication rates.

**RESULTS:**

A total of 144 patients were included, with 72 undergoing vaginal natural orifice transluminal endoscopic surgery and 72 undergoing vaginal hysterectomy. No significant differences were found in demographic characteristics, surgical duration (100.8±14.1 min for vaginal natural orifice transluminal endoscopic surgery vs. 99.3±26.7 min for vaginal hysterectomy, p=0.646), or complication rates. The vaginal natural orifice transluminal endoscopic surgery group had a higher rate of bilateral salpingo-oophorectomy (97.2 vs. 36.1%, p<0.001) and required fewer non-steroidal anti-inflammatory drugs postoperatively (median 2.0 vs. 3.0, p=0.003).

**CONCLUSION:**

This study indicates that vaginal natural orifice transluminal endoscopic surgery is a feasible alternative to vaginal hysterectomy for benign hysterectomies, demonstrating comparable outcomes and potential benefits in postoperative pain management. Future multi-center studies are warranted to strengthen the evidence base and explore long-term outcomes.

## INTRODUCTION

Hysterectomy is one of the most commonly performed surgical procedures for benign gynecological conditions^1^. There are two main surgical approaches: laparotomy and minimally invasive surgery, which include vaginal, laparoscopic, robot-assisted, and laparoscopically-assisted techniques. Vaginal hysterectomy (VH) is the preferred surgical route due to its favorable postoperative outcomes, lower complication rates, and potential for outpatient procedures, leading to increased patient satisfaction and reduced healthcare costs^2-4^.

Natural Orifice Transluminal Endoscopic Surgery (NOTES) is an innovative minimally invasive technique that allows surgical procedures through natural orifices, minimizing visible scarring and potentially reducing postoperative pain. Vaginal natural orifice transluminal endoscopic surgery (vNOTES), a specific variant designed for hysterectomy, has shown feasibility and safety, offering advantages such as a scar-free abdomen and improved access to the adnexa, which may enhance outpatient management^5^.

However, there is a lack of rigorous randomized controlled trials directly comparing vNOTES with conventional VH, the current standard for benign hysterectomies. Therefore, this study aims to evaluate and compare the surgical outcomes of vNOTES and VH, addressing a critical gap in the literature on surgical methodologies for benign gynecological conditions.

## METHODS

### Study design and setting

This prospective randomized study comparing vaginal hysterectomy and vNOTES hysterectomy for benign indications was conducted at a tertiary hospital between January 2025 and February 2025. The study protocol was developed by the principles outlined in the Declaration of Helsinki and received approval from the Research Ethics Committee (Approval number: 2021/514/214/25, date: 30.11.2021). The study was registered with ClinicalTrials.gov (NTC06776718).

### Participant selection

The inclusion criteria for this study were patients aged between 40 and 80 years who had undergone hysterectomy for benign gynecological conditions. Patients who had undergone urogynecological procedures (including pelvic organ prolapse surgery and mid-urethral sling procedures), those with suspected gynecologic malignancies, endometriosis, tubal-ovarian abscesses, pelvic organ prolapse exceeding grade 2, individuals who refused to participate in the study, and patients whose medical records could not be obtained were excluded from the study.

The two-sided test was adopted, setting the level of significance alpha at 5% (a=0.05) and the power of the sample at 80% (1-β=0.80). According to preliminary assumptions, a medium effect size (Cohen's d=0.5) was considered clinically meaningful to detect differences in total surgery duration between the vNOTES and vaginal hysterectomy groups. Based on this, the minimum required sample size was calculated as N1=N2=64 cases using G*Power 3.1 software (Heinrich Heine University, Düsseldorf, Germany).

To ensure statistical robustness and account for potential dropouts, 72 participants were recruited in each group, totaling 144 patients. In the final analysis, the mean total surgery duration was 100.8±14.1 min for the vNOTES group and 99.3±26.7 min for the vaginal hysterectomy group.

### Randomization and intervention

Eligible participants were randomly assigned to undergo either vaginal hysterectomy or vNOTES hysterectomy for benign indications using a computer-generated randomization scheme. The opaque sealed envelope was opened by an independent operating room nurse to reveal the surgical technique to be used.

The surgical technique for conventional VH has been comprehensively detailed in a prior publication^6^. All vNOTES hysterectomy procedures were conducted as vaginally assisted vNOTES. We adhered to the surgical techniques for vNOTES procedures as previously reported^7^. All surgical procedures were performed by or under the direct supervision of surgeons with extensive experience in minimally invasive techniques.

### Outcomes

The primary outcome of this study was the duration of the surgical procedure, and the secondary outcomes included the visual analog scale (VAS) score and the rate of intraoperative and postoperative complications. Data on sociodemographic and clinical characteristics, and surgical details, were extracted from hospital medical records and patient files. The sociodemographic and clinical data encompassed variables such as age, body mass index, parity, and history of previous abdominal surgeries. Surgical data included operative time, length of hospital stay, intraoperative and postoperative complications, readmission rates within the first month following surgery, pain scores recorded at 6 and 24 h postoperatively using a VAS, which ranges from 0 (indicating no pain) to 10 (indicating the worst pain imaginable), and the total amount of postoperative analgesics administered. The surgical operative time was defined as the interval from the initial incision to the completion of vaginal cuff closure, known as the "cut-to-close" time. Uterine volume was quantified in milliliters based on measurements of the pathological specimens, calculated using the formula: Uterine volume (mL)=(anterior-posterior diameter)×(sagittal diameter)×(transverse diameter)×0.0005236.

### Statistical analysis

All statistical analyses were performed using IBM SPSS version 20.0 software (SPSS Inc., Armonk, NY, USA). Continuous variables are presented as means±standard deviations (SD), whereas categorical variables are expressed as counts and percentages. The Kolmogorov-Smirnov test was utilized to assess the normality of the data distribution. The Student's t-test was employed for group comparisons, while the chi-square test was used for the comparison of dichotomous variables. The paired samples t-test was applied to compare preoperative and postoperative data sets. A p-value of less than 0.05 was considered statistically significant.

## RESULTS

**Figure 1:**
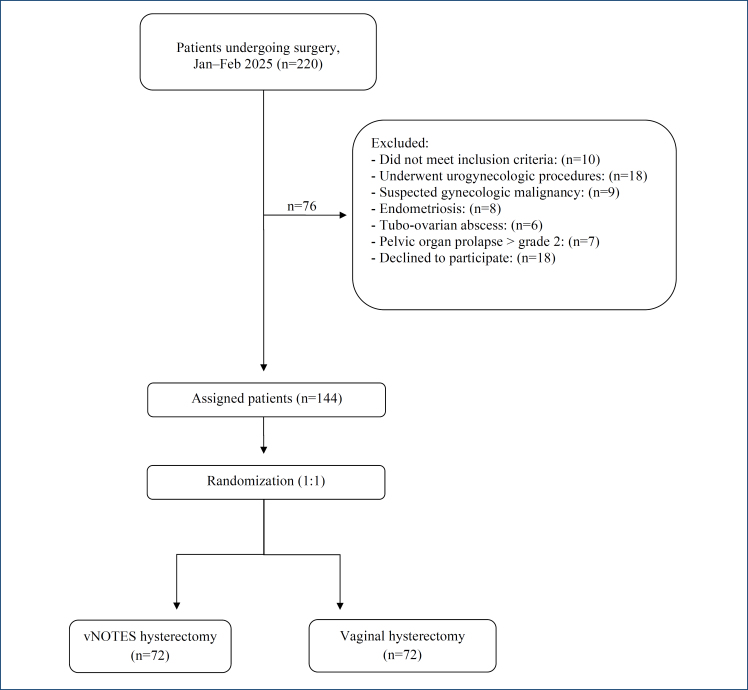
Flow diagram of the study.

A total of 220 patients who underwent gynecologic surgery between January and February 2025 were assessed for eligibility. Of these, 76 patients were excluded based on predefined criteria. Specifically, 10 patients did not meet the inclusion criteria, 18 had previously undergone urogynecological procedures, nine were suspected of having gynecologic malignancy, eight had endometriosis, six presented with tubo-ovarian abscesses, seven had pelvic organ prolapse exceeding grade 2, and 18 were excluded due to refusal to participate to the study. Following exclusion, 144 patients were deemed eligible and were randomly assigned to one of two study arms. Seventy-two patients were allocated to the vNOTES hysterectomy group, and seventy-two patients were allocated to the VH group. A total of 144 patients were included in the final analysis (Figure 1).

No statistically significant differences were identified between the two groups concerning age (p=0.336), body mass index (p=0.868), parity (p=0.092), menopausal status (p=0.190), or comorbidity (p=0.388). A history of prior abdominal surgery was present in 29.8% (n=23) of patients in the vNOTES group, compared to 34.7% (n=25) in the VH group. Statistical analysis indicated no significant differences between the groups with regard to the surgical indications (p=0.737) or the history of previous abdominal surgeries (p=0.684). The demographic characteristics of both groups are detailed in Table 1.

**Table 1:** Patient characteristics.

		vNOTES hysterectomy group (n=72)	Vaginal hysterectomy group (n=72)	p-value
Age (year, mean±SD)		58.02±9.18	59.27±8.78	0.336
BMI (kg/m^2^), mean±SD		28.02±4.89	28.13±4.03	0.868
Parity, (median±IQR)		3.5±2.75	4±2	0.092
Post-menopausal, n (%)		60 (83.3)	64 (88.8)	0.190
Co-morbidities, n (%)		41 (56.9)	36 (50)	0.388
	● Hypertension, n (%)	24 (33.3)	21 (29.1)	
	● Diabetes mellitus, n (%)	13 (18)	11 (15.2)	
	● Coronary artery disease, n (%)	5 (6.9)	4 (5.5)	
	● Asthma, n (%)	6 (8.3)	5 (6.9)	
	● Hypothyroidism, n (%)	9 (12.5)	8 (11.1)	
Operation indication				
	● Adenomyosis, n (%)	7 (9.7)	8 (9.6)	0.737
	● Medical treatment resistant abnormal uterine bleeding, n (%)	31 (43)	29 (40.3)	
	● Uterine leiomyoma, n (%)	8 (11.1)	10 (13.8)	
	● Endometrial hyperplasia without atypia, n (%)	26 (36.1)	25 (34.7)	

BMI: body mass index; vNOTES: transvaginal natural orifice transluminal endoscopic surgery; SD: standard deviation; IQR: ınterquartile range.

In the vNOTES hysterectomy group, 70 patients (97.2%) underwent bilateral salpingo-oophorectomy, compared to 26 patients (36.1%) in the VH group, a statistically significant difference (p<0.001). The uterine volumes between the two groups were comparable (vNOTES group 0.125±0.10, VH group 0.129±0.08, p=0.788).

There was no difference between vNOTES and VH groups in terms of re-admission rates (n=72, 5.5% for vNOTES; n=72, 2.8% for VH; p=0.079), mean surgical duration (100.8±14.1 min for vNOTES; 99.3±26.7 min for VH; p=0.646), median hospitalization time (43.09±14.8 h for vNOTES; 41.13±16.0 h for VH; p=0.080), and mean decrease in hematocrit levels (3.91±3.0 g/dL for vNOTES; 3.48±1.5 g/dL for VH; p=0.163).

Both groups had a bladder injury rate of 1.4% (n=1, p=1.000). The vNOTES group experienced a higher postoperative ileus rate of 4.2% (n=3) compared to 2.8% (n=2) in the VH group (p=0.638). Vaginal bleeding was reported in 1.4% (n=1) of the vNOTES group and 2.8% (n=2) of the VH group (p=0.638).

The mean VAS pain scores were 5.5±1.0 for the vNOTES group and 6.0±1.0 for the VH group six hours after surgery (p=0.233). At 24 h, the scores were similar, with the vNOTES group at 2.0±2.0 and the VH group at 2.0±0.0 (p=0.111).

The vNOTES group used fewer non-steroidal anti-inflammatory drugs (NSAIDs) (median 2.0±1.0) than the VH group (3.0±1.0, p=0.003), with both groups having a 0% requirement for narcotic analgesics (Table 2).

**Table 2:** Surgical outcomes, pain scores, analgesics requirements, and intra-postoperative complications.

		vNOTES hysterectomy group (n=72)	Vaginal hysterectomy group (n=72)	p-value
Duration of surgery, min (mean±SD, min–max)		100.8±14.1	99.3±26.7	0.646
Hospitalization time, h (median±IQR, min–max)		43.09±14.8	41.13±16.0	0.080
Re-admission, n (%)		4 (5.5)	2 (2.8)	0.079
Drop in hematocrit levels, % (mean±SD, min–max)		3.91±3	3.48±1.5	0.163
Intraoperative complications, n (%)				
	● Bladder injury	1 (1.4)	1 (1.4)	1
Postoperative complications, n (%)		3 (4.2)	2 (2.8)	0.638
	● Postoperative ileus, n (%)	2 (2.8)	–	
	● Vaginal bleeding, n (%)	1 (1.4)	2 (2.8)	
VAS score at postoperative 6th hour, (median±IQR)		5.5±1	6±1	0.233
VAS score at postoperative 24th hour, (median±IQR)		2±2	2±0	0.111
Total doses of NSAİD analgesics used, (median±IQR)		2±1	3±1	0.003*
Narcotic analgesics requirement, n (%)		0	0	NA

NA: not applicable; vNOTES: transvaginal natural orifice transluminal endoscopic surgery; VAS: visual analog scale; SD: standard deviation; NSAID: non-steroidal anti-ınflammatory drug; IQR: interquartile range. Continuous variables are reported as mean±standard deviation or median (first quartile to third quartile) depending on distribution characteristics. Binary variables are represented as count (percentage).

* p<0.05 statistically significant.

## DISCUSSION

This study compared vNOTES and VH for benign hysterectomies and found no significant differences in surgical outcomes between the two techniques. Notably, the vNOTES group exhibited a higher rate of salpingectomy or adnexectomy. Additionally, the vNOTES group required fewer NSAIDs compared to the VH group.

A retrospective cohort study comparing vNOTES hysterectomy with conventional VH for benign conditions revealed no significant differences in outcomes, which aligns with our findings^8^. The authors noted that the vNOTES group exhibited slightly higher rates of adnexectomy and salpingectomy, which are crucial for mitigating future ovarian malignancy risks^9^. Although these interventions are essential, they can pose technical challenges in the context of conventional VH. This may be attributed to the enhanced visualization and access provided by vNOTES, which could facilitate the execution of these procedures^10,11^. Tekin et al.^12^ further support these advantages by demonstrating that a "vNOTES-first" approach is feasible and safe across a wide range of benign gynecologic surgeries, including cases with enlarged uteri or prior abdominal surgeries, highlighting its versatility and clinical efficacy.

A recent systematic review and meta-analysis comparing vNOTES and VH for benign indications found no significant differences in surgical and postoperative outcomes, including operation duration, blood loss, hospital stay, VAS pain scores one day postoperatively, and complication rates^13^. However, vNOTES offered advantages such as improved visualization, better cosmetic results, and reduced tissue trauma^14,15^. Consistent with these findings, our study also found no significant differences in VAS pain scores between the vNOTES and VH groups, indicating comparable levels of pain relief. The vNOTES group showed a reduced need for NSAIDs, likely due to lower tissue trauma, which may enhance recovery.

The VH and the vNOTES technique offer several advantages, including a reduced need for blood transfusions, lower postoperative pain, quicker recovery, the absence of abdominal wall complications, and enhanced aesthetic outcomes^3,5,14^. In addition, a recent reseach has emphasized that vaginal routes, both VH and vNOTES, represent significantly greener alternatives to laparoscopy by reducing reliance on disposable instruments and lowering the surgical carbon footprint, thereby aligning gynecologic practice with principles of environmental sustainability^16^. However, it is important to acknowledge that vNOTES is a relatively expensive technique. In low-resource settings, VH may be a more feasible and cost-effective alternative, providing similar benefits while alleviating the financial burden associated with vNOTES.

Consistent with our findings, the randomized trial by Tormena et al.^17^, which compared single-port and multiport laparoscopic hysterectomies, demonstrated comparable inflammatory responses despite a longer operative duration in the single-port group. Similarly, while vNOTES may require additional setup time, it achieves equivalent postoperative outcomes to VH with respect to pain, complications, and recovery. These parallels support the incorporation of vNOTES within minimally invasive surgery, emphasizing its potential for reduced tissue trauma, expedited recovery, and minimized analgesic use.

### Strengths and limitations

A major strength of our study lies in its prospective, randomized controlled design, which confers robust internal validity and mitigates selection and performance biases. Moreover, this investigation represents the first randomized controlled trial conducted in Turkey comparing vNOTES and VH for benign indications, thereby addressing a notable gap in the existing literature. All procedures were conducted by an experienced team of surgeons with advanced proficiency in minimally invasive gynecologic techniques within a high-volume tertiary care setting, further ensuring procedural consistency and reliability of outcomes. However, several limitations must be acknowledged. The single-center design may constrain the generalizability of our findings to broader populations and practice settings. Additionally, the relatively short follow-up interval precluded the assessment of longer-term outcomes, such as pelvic floor function, sexual health, and health-related quality of life. The study also lacked biomarker-based assessments of surgical stress and inflammatory response, which could have enriched our comparative evaluation of physiologic recovery. Furthermore, patient-reported outcome measures (PROMs) were not included, limiting our ability to evaluate subjective recovery experiences. Future multicenter trials with extended follow-up and incorporation of PROMs and biologic endpoints are warranted to comprehensively assess the longitudinal efficacy and patient-centered benefits of vNOTES relative to VH.

### Clinical ımplications

For gynecologic surgeons and healthcare providers involved in procedural planning for benign uterine pathology, our findings affirm that vNOTES is a safe and effective alternative to conventional VH. Its utility is particularly notable in patients requiring adnexal intervention, where enhanced visualization and access conferred by the endoscopic platform may offer clinical advantages. The decision between vNOTES and VH should be individualized, guided by patient anatomy, comorbid conditions, surgical complexity, and institutional expertise. In resource-rich environments with adequate training and equipment, vNOTES may represent an evolution of minimally invasive hysterectomy with added value in terms of cosmesis, access to adnexa, and analgesic sparing.

## CONCLUSION

The findings of this study indicated that both the vNOTES and VH techniques yielded comparable surgical outcomes. Although vNOTES showed promise in reducing postoperative analgesic requirements, both approaches demonstrated comparable safety profiles. Future research should prioritize multicenter studies to strengthen the findings and investigate long-term patient satisfaction and quality of life. These efforts will be crucial for optimizing surgical strategies in the management of benign gynecological conditions.

## DECLARATION OF HELSINKI

The database management is in accordance with privacy legislation, and the presented study is in accordance with the ethical principles of the Declaration of Helsinki. Ethical approval for this study was obtained by the Institutional Scientific Research Ethical Board (Approval number: 2021/514/214/25). Written and verbal informed consent was obtained from all study participants. It has been registered on ClinicalTrials.gov. (ref. no: NCT06776718) and complied with the Consolidated Standards of Reporting Trials (CONSORT) guidelines.

## Data Availability

The datasets generated and/or analyzed during the current study are available from the corresponding author upon reasonable request.
